# CT-guided Thermal Ablation of Postoperative Isolated Bile Leakages—Evaluation of Safety and Therapeutic Effectiveness

**DOI:** 10.1007/s00270-025-04278-2

**Published:** 2025-12-07

**Authors:** Clarissa Hosse, Johannes Kolck, Felix Krenzien, Timo A. Auer, Dominik Geisel, Wenzel Schöning, Bernhard Gebauer, Uli Fehrenbach

**Affiliations:** 1 Department of Radiology, Charité – Universitätsmedizin Berlin, Augustenburger Platz 1, 13353 Berlin, Germany; 2 Department of Surgery CCM / CVK, Charité – Universitätsmedizin Berlin, Berlin, Germany

**Keywords:** Bile leakage, Thermal ablation, CT-guided intervention, Disconnected bile ducts, Postoperative complication, Interventional radiology, Hepatic surgery, Biloma, Non-surgical management

## Abstract

**Purpose:**

To describe the technique and evaluate the safety and clinical efficacy of CT-guided thermal ablation for postoperative isolated bile leakage (IBL) in patients with disconnected bile ducts.

**Materials and Methods:**

This retrospective study included 14 patients with postoperative IBL following liver resection between 2016 and 2024. All patients underwent CT-guided radiofrequency ablation (RFA) as treatment for IBL. Technical success was defined as appropriate coverage of the leakage site on post-ablation CT. Clinical success was defined as cessation of IBL; time to leak cessation was recorded accordingly. Total percutaneous biloma drainage time and peri-/post-interventional complications were evaluated accordingly.

**Results:**

Technical success was achieved in all ablation procedures with no major adverse events. Clinical success was observed in 93% (*n* = 13) of patients. One patient experienced recurrence of IBL within 30 days. Median total drainage time was 33.5 (IQR 20–62) days. The median time to leak cessation after RFA was 4.5 (IQR 4–6) days.

**Conclusion:**

CT-guided thermal ablation appears to be an effective and safe treatment option for postoperative IBL, helping to reduce the duration of drainage therapy.

**Graphical Abstract:**

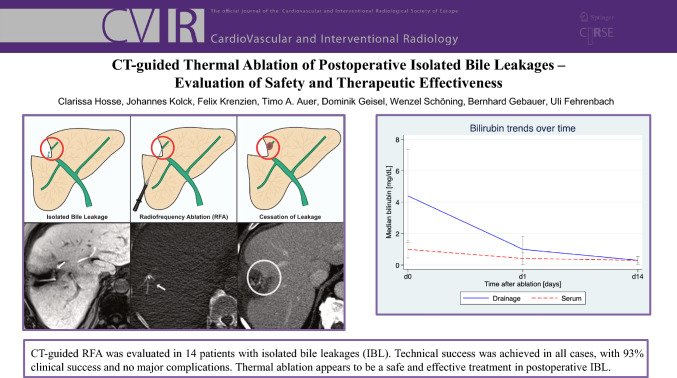

## Introduction

Postoperative bile leakage (PBL) is a frequent and potentially severe complication following liver surgery, with reported incidence rates between 4 and 10% [1–4].

Most bile leaks originate from peripheral bile ducts and can be managed successfully with conservative measures, percutaneous biloma drainage (PBD), endoscopic retrograde cholangiopancreatography (ERCP) with biliary stenting or papillotomy, or percutaneous transhepatic biliary drainage (PTBD) [5]. These strategies are generally effective when the leaking duct remains in continuity with the central biliary tree. In contrast, isolated bile leakage (IBL) from disconnected ducts represents a particularly challenging entity. These ducts no longer communicate with the main biliary system, rendering standard biliary approaches ineffective [6–8]. In such cases, long-term PBD is often the only feasible conservative strategy. Redo surgery with segmental resection has been advocated in selected cases, but is associated with high morbidity and mortality. Chemical ablation using ethanol, acetic acid, or other sclerosing agents has been proposed as a minimally invasive treatment option for IBL [9]. Chemical ablation is technically demanding, success rates vary, and treatment failure often necessitates repetitive procedures [10]. Given these limitations, there is a need for alternative minimally invasive options. Percutaneous thermal ablation is an established technique in interventional oncology and offers precise, image-guided induction of local necrosis [11]. Applied to IBL, radiofrequency ablation (RFA) may seal the disconnected duct by creating localized necrosis and fibrosis of the liver parenchyma at the leakage site. Our rationale for applying thermal ablation was derived from the surgical principle of cauterizing a biliary leakage at the resection margin intraoperatively. In this sense, thermal ablation represents a minimally invasive, percutaneous analog of this established intraoperative technique. The potential advantages could include single-session treatment, direct targeting under CT guidance, and avoidance of chemical irritants. In this technical note, we report our experience with CT-guided thermal ablation for IBL, focusing on technical feasibility, safety, and early clinical efficacy.

## Material and Methods

### Study Design and Patient Cohort

This retrospective study was approved by our institutional ethics committee (EA1/176/24). Written informed consent was obtained from all patients. Between 2016 and 2024, 14 patients with postoperative IBL underwent CT-guided thermal ablation (Table 1).

**Table 1:** Patient characteristics

Median age (years)	63.5 (IQR: 45–73)
Gender	
Male (%)	64 (*n* = 9)
Female (%)	35 (*n* = 5)
Primary disease	
Hepatocellular carcinoma (%)	21 (*n* = 3)
Intrahepatic cholangiocarcinoma (%)	29 (*n* = 4)
Perihilar cholangiocarcinoma (%)	29 (*n* = 4)
Liver metastases (%)	21 (*n* = 3)
Surgery	
Right HH (%)	36 (*n* = 5)
Right trisectionectomy (%)	14 (*n* = 2)
Left HH (%)	14 (*n* = 2)
Left trisectionectomy (%)	7 (*n* = 1)
Non-anatomical resection (%)	29 (*n* = 4)

### PBL Treatment Algorithm

If PBL was clinically suspected (abdominal pain, increasing infection parameters, elevated bilirubin in drainage fluids [12]), imaging was performed to rule out or to confirm a possible biloma. If a collection was found to be suspicious of a biloma that was not addressed by an intraoperatively inserted drain, a PBD was inserted, and the drainage fluid was analyzed for the presence of bile. In case of PBL and continuous bile leakage after PBD insertion, all endoscopically accessible patients were then examined with ERCP, and in case of a confirmed peripheral or central PBL, endoscopic treatment was performed in the same session. If there was no visible PBL on ERCP or the biliary tree was not accessible endoscopically, a Gd-EOB-enhanced MRI was performed. This examination technique was used to confirm the active PBL by extravasation of contrast medium into the collection and to localize the leakage site. In cases where a disconnected duct and IBL could be detected in Gd-EOB MRI, CT-guided thermal ablation of the leakage was planned accordingly. All patients received intravenous antibiotic therapy peri-interventional (standard regimen with tazobactam; adjusted according to microbiological findings and resistance testing, if available).

### Procedural Technique

All ablation procedures were performed by board-certified interventional radiologists in a large university hospital with extensive experience in CT-guided thermal ablation. A total of four interventional radiologists performed the procedures.

All procedures were conducted using the same CT scanner (Somatom Definition AS, Siemens Healthineers, Erlangen, Germany). To plan the puncture tract, a non-contrast-enhanced CT scan (120 kVp, automatic tube current modulation) of the upper abdomen was acquired in the supine position, ensuring complete liver coverage. This planning CT was visually co-registered with Gd-EOB-MRI for leakage localization and trajectory optimization.

**Fig. 1:**
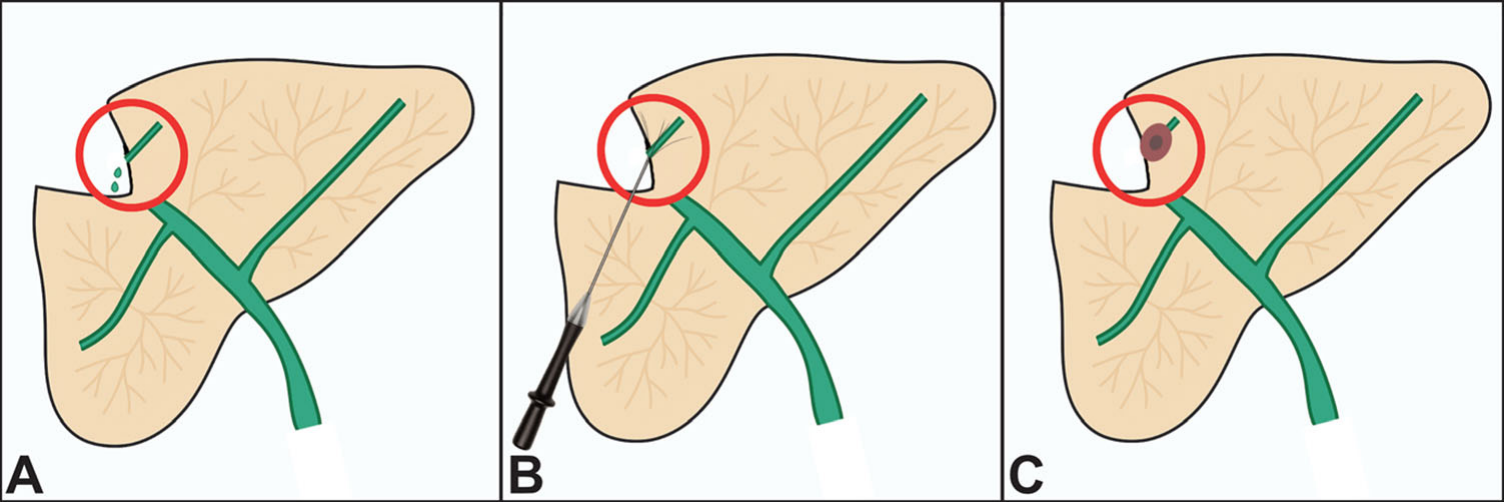
**A**: Identification of an isolated bile leakage by a disconnected duct (in hepatocyte-specific contrast-enhanced MRI with active biliary leakage). **B**: CT-guided placement of the radiofrequency ablation probe at the site of leakage. **C**: Confirmation of the ablation zone and consequently cessation/reduction of the isolated bile leakage

Monopolar radiofrequency ablation (RFA) was performed using a RITA StarBurst SemiFlex system (AngioDynamics, Latham, NY, USA), consisting of a generator operating at a frequency of 350–500 kHz. Following the acquisition of the non-contrast CT scan, the ablation probe was advanced under CT fluoroscopy guidance toward the IBL. The ablation target was the leaking site located at the hepatic resection margin. The umbrella-shaped electrodes were deployed approximately 2 cm beyond the tip to encompass the target area. Upon satisfactory positioning, ablation was initiated with a target temperature of 90–110 °C, maintained for approximately 10 min at a maximum power output of 100–200 watts. Following the completion of the procedure, the applicator was carefully retracted, and a contrast-enhanced CT scan was performed to assess ablation coverage and exclude immediate complications. The procedure is illustrated in Fig. 1. All procedures were carried out under local anesthesia combined with deep conscious sedation.

### Definitions and Data

Technical success was defined as an appropriate ablation zone at the leakage site in the post-ablation contrast-enhanced CT scan. Clinical success was defined as cessation of leakage or a decrease in the bilirubin concentration in the drainage secretions of the PBD to below or to the level of systemic values. Patients with persistent bilious secretion via the PBD (observation period of 14 days) or patients who received a new drainage (within 30 days of drain removal) with evidence of continuous bile leakage were defined as treatment failures. Bilirubin was determined in the serum and in the drainage at the day of ablation (d0), the day after the procedure (d1) and 12–14 days (d14) after the intervention (or last possible, if the leakage had ceased before). The bilirubin ratio (bilirubin_Drainage_/bilirubin_Serum_) and time to leak cessation were recorded accordingly. Total drainage time was defined as the interval between initial placement of a drainage catheter and definitive removal of all drains. Data were collected from electronic patient charts, the radiology information system (RIS), and the picture archiving and communication system (PACS). Complications were evaluated according to the CIRSE Classification System with an observation time of 30d after drain removal [13].

### Statistics

Normality of the data was assessed using the Shapiro–Wilk test and was not confirmed (*p* < 0.05); therefore, nonparametric statistical tests were applied for all subsequent analyses. Continuous variables were presented as median and interquartile range (IQR; 25th–75th percentile). *P* values below 0.05 were considered statistically significant.

## Results

### Ablation Procedure and Postprocedural Outcome

IBL was typically diagnosed within one to four weeks postoperatively. Technical success was achieved in all 14 procedures (100%). In four cases, two adjacent ablation zones were chosen by the operating interventional radiologist to increase the likelihood of effectively targeting the leakage site. Clinical success was observed in 13/14 patients (93%). One patient experienced recurrence of bile leakage within 30 days, which was successfully managed with PBD (Table 2). No major complications occurred.

**Table 2:** Procedure characteristics and outcome overview of CT-guided thermal ablations of isolated bile leakages

Procedure specifications (temperature, duration)	Radiation exposure (mGy*cm)	Number of ablation zones	Technical success, clinical success
100 °C, 7 min	576	2	yes, yes
105 °C, 10 min	334	1	yes, yes
110 °C, 10 min	585	1	yes, yes
95 °C, 8 min	406	1	yes, yes
95 °C, 8 min	242	1	yes, yes
105 °C, 10 min	805	1	yes, no
105 °C, 20 min	525	2	yes, yes
105 °C, 10 min	1579	2	yes, yes
105 °C, 10 min	823	1	yes, yes
100 °C, 8 min	733	2	yes, yes
90 °C, 10 min	701	1	yes, yes
95 °C, 8 min	531	1	yes, yes
110 °C, 10 min	347	1	yes, yes
100 °C, 8 min	745	1	yes, yes
90 °C, 10 min	415	1	yes, yes

**Fig. 2:**
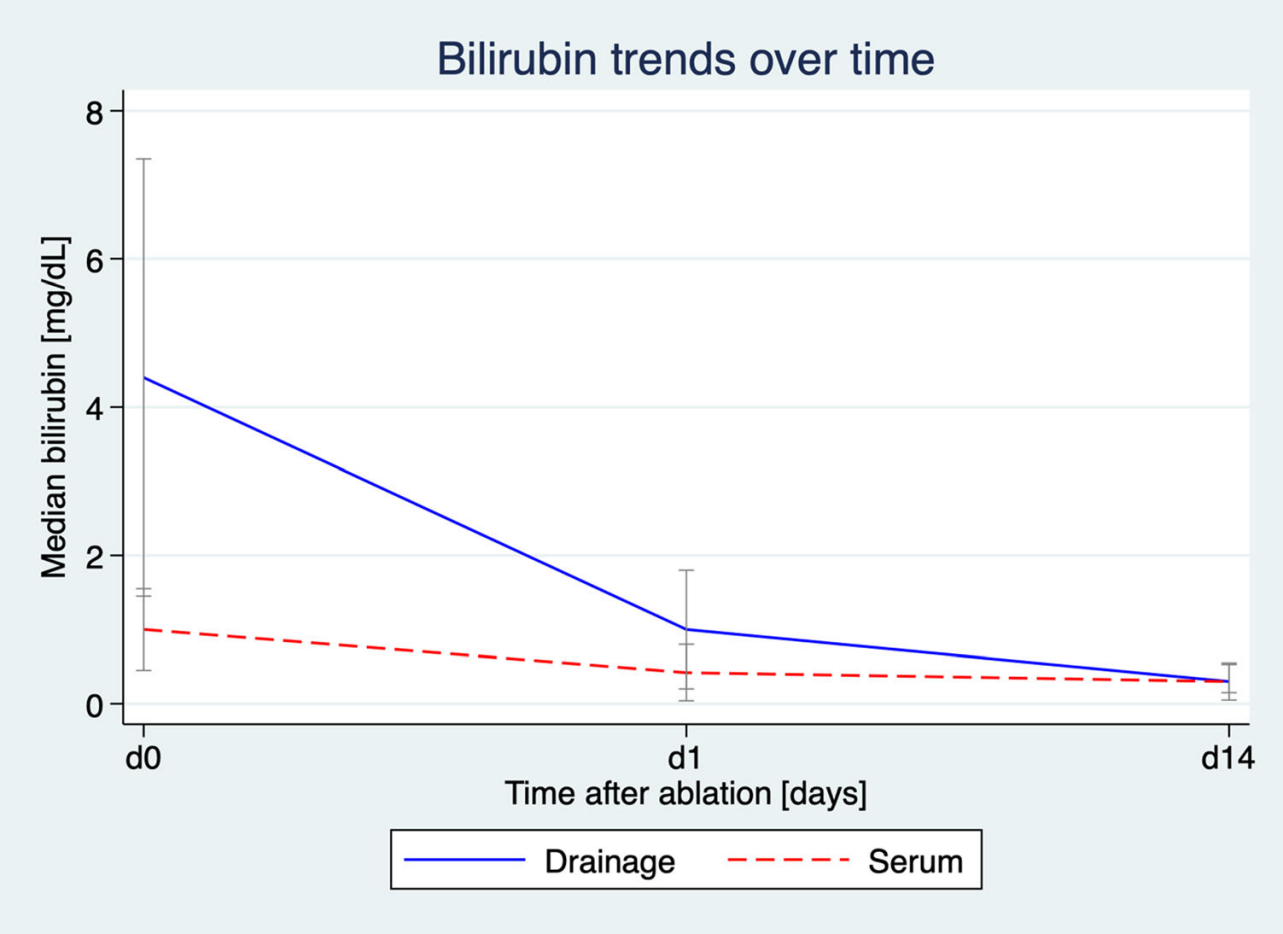
Serum and drainage bilirubin dynamics in the ablation group. Median serum and drainage bilirubin levels + IQR (mg/dL) over time (D0, D1, D14) in patients treated with CT-guided thermal ablation. A steady decline in drainage bilirubin indicates effective local control of the isolated bile leakages

**Fig. 3:**
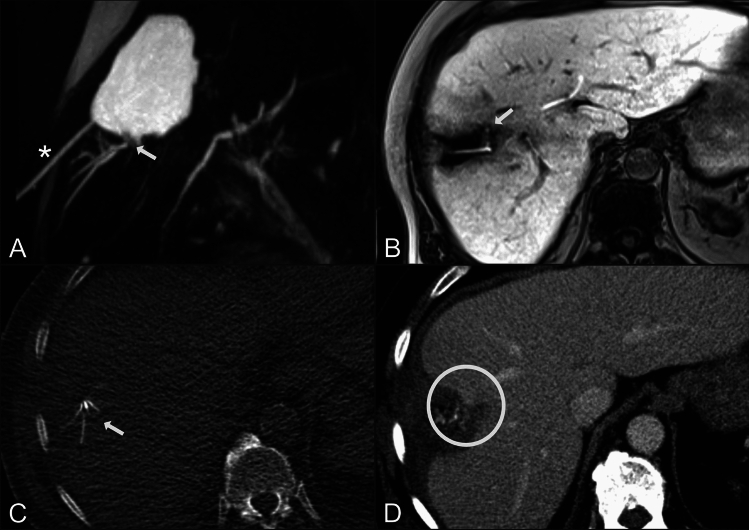
**A**: Pre-interventional T2w-MRCP displaying a disconnected duct (arrow) emptying into a biloma, which is already drained by a percutaneous biliary drainage (asterisk). **B**: Hepatocyte-specific contrast-enhanced MRI demonstrates contrast extravasation into the biloma (arrow), providing non-invasive evidence of an active bile leak. **C**: CT fluoroscopy demonstrates correct placement of the extended radiofrequency ablation probe (arrow) at the leakage site. **D**: Post-ablation contrast-enhanced CT demonstrates the ablation zone at the leakage site of the disconnected duct (circle). In this patient, the isolated bile leakage completely ceased six days after the ablation

Median values of bilirubin in the drainage and serum were markedly elevated pre-intervention and showed a large decrease after ablation as shown in Fig. 2. The median bilirubin ratio (bilirubin_Drainage_/bilirubin_Serum_) dropped from 4.31 (IQR 2.56–7.81) at the day of biliary ablation (d0) to 2.1 (IQR 0.9–4.42) at the first post-interventional day (d1) to 1.03 (IQR 0.49–1.76) at d14. The Friedman test demonstrated significant differences in measurements across the three time points (Friedman *χ*^2^ = 22.37, Kendall’s *W* = 0.62, *p* = 0.034). The median time to leak cessation was 4.5 (IQR 4–6) days following the ablation procedure. The median total drainage time was 33.5 (IQR 20–62) days. Figure 3 illustrates the procedure using a representative case example.

## Discussion

Our study demonstrates that CT-guided thermal ablation represents a technically feasible, safe, and clinically effective option for the management of postoperative IBL. Treatment of IBL is challenging, as the lack of continuity with the central biliary system precludes endoscopic interventions or PTBD. In our small series, ablation resulted in rapid cessation of IBL in most patients, without any major complications.

Several alternative strategies have been proposed for IBL management. Long-term PBD remains the most applied approach, but it is associated with prolonged hospitalization, impaired quality of life, and an increased risk of recurrent infections [14]. Surgical resection of the affected segment can provide definitive treatment but is rarely feasible due to patient frailty and the high morbidity [8]. Previous reports on IBL have emphasized the limited treatment options and the need for alternatives to revision surgery such as chemical sclerotherapy [6]. Chemical ablation using ethanol or N-butyl cyanoacrylate is the most widely used advanced minimally invasive alternative. While success has been reported, these techniques are technically demanding, often require multiple treatment sessions, and may cause severe adverse events such as chemical peritonitis [9, 10, 15].

In contrast, CT-guided thermal ablation could offer several advantages. The procedure can usually be performed in a single session, directly targets the leakage site, and induces local necrosis that functionally seals the disrupted duct [16]. Integration of Gd-EOB-enhanced MRI into pre-procedural planning was critical in our workflow, as it enabled precise localization of the leaking duct and optimized probe placement. Moreover, the umbrella-shaped electrode design allowed selective coverage of the leakage site while sparing adjacent vasculature or other critical structures as biliary enteric anastomoses. These technical aspects may explain the high success rate and absence of major complications in our cohort.

This study is limited by its retrospective design, small sample size, and single-center setting. Future studies are warranted to validate our findings and to better define patient selection criteria and long-term outcomes. A further limitation of our study is the unknown long-term fate of the ablated liver segments. While short-term technical and clinical efficacy could be demonstrated, the long-term evolution of ablated parenchymal areas remains unclear and warrants further investigation. At our institution, only RFA was employed for thermal ablation of IBL, whereas microwave ablation (MWA) may represent a comparable alternative that warrants further investigation.

In conclusion, CT-guided thermal ablation represents a promising therapeutic strategy for postoperative IBL, combining high clinical efficacy with low complication rates, and should be considered an early interventional option to avoid prolongation of drainage therapy.

## Abbreviations

PBL: Postoperative bile leakage
IBL: Isolated bile leakage
CT: Computed tomography
RFA: Radiofrequency ablation
MRI: Magnetic resonance imaging
Gd-EOB: Gadolinium ethoxybenzyl diethylenetriamine pentaacetic acid
ERCP: Endoscopic retrograde cholangiopancreatography
PBD: Percutaneous biloma drainage
PTBD: Percutaneous transhepatic biliary drainage
HH: Hemihepatectomy
